# Quantitative X-ray Computed Tomography Peritoneography in Malignant Peritoneal Mesothelioma Patients Receiving Intraperitoneal Chemotherapy

**DOI:** 10.1245/s10434-013-2976-8

**Published:** 2013-05-24

**Authors:** Joshua C. Leinwand, Binsheng Zhao, Xiaotao Guo, Saravanan Krishnamoorthy, Jing Qi, Joseph H. Graziano, Vesna N. Slavkovic, Gleneara E. Bates, Sharyn N. Lewin, John D. Allendorf, John A. Chabot, Lawrence H. Schwartz, Robert N. Taub

**Affiliations:** 1 Division of Medical Oncology, Department of Medicine, Columbia University Medical Center, New York, NY USA; 2 Department of Radiology, Columbia University Medical Center, New York, NY USA; 3 Department of Environmental Health Sciences, Columbia University Medical Center, New York, NY USA; 4 Division of Gynecologic Oncology, Department of Obstetrics and Gynecology, Columbia University Medical Center, New York, NY USA; 5 Department of Surgery, Columbia University Medical Center, New York, NY USA

## Abstract

**Background:**

Intraperitoneal chemotherapy is used to treat peritoneal surface-spreading malignancies. We sought to determine whether volume and surface area of the intraperitoneal chemotherapy compartments are associated with overall survival and posttreatment glomerular filtration rate (GFR) in malignant peritoneal mesothelioma (MPM) patients.

**Methods:**

Thirty-eight MPM patients underwent X-ray computed tomography peritoneograms during outpatient intraperitoneal chemotherapy. We calculated volume and surface area of contrast-filled compartments by semiautomated computer algorithm. We tested whether these were associated with overall survival and posttreatment GFR.

**Results:**

Decreased likelihood of mortality was associated with larger surface areas (*p* = 0.0201) and smaller contrast-filled compartment volumes (*p* = 0.0341), controlling for age, sex, histologic subtype, and presence of residual disease >0.5 cm postoperatively. Larger volumes were associated with higher posttreatment GFR, controlling for pretreatment GFR, body surface area, surface area, and the interaction between body surface area and volume (*p* = 0.0167).

**Discussion:**

Computed tomography peritoneography is an appropriate modality to assess for maldistribution of intraperitoneal chemotherapy. In addition to identifying catheter failure and frank loculation, quantitative analysis of the contrast-filled compartment’s surface area and volume may predict overall survival and cisplatin-induced nephrotoxicity. Prospective studies should be undertaken to confirm and extend these findings to other diseases, including advanced ovarian carcinoma.

Catheter-administered outpatient intraperitoneal (IP) chemotherapy has been used for peritoneal surface-spreading malignancies to maximize local drug concentrations for longer durations than possible with systemic therapy.^1^,^2^ This rationale is supported by pharmacokinetic studies describing the “pharmacokinetic advantage” of IP administration of various drugs, i.e., the ratio of intraperitoneal to intravascular drug levels, expressed either in peak concentrations or areas under the time-concentration curve (AUC).^3^–^15^

X-ray computed tomography (CT) peritoneography has been previously used in patients receiving catheter-administered outpatient IP chemotherapy to assess for catheter failure and infusate maldistribution.^16^–^20^ However, outcomes data from patients assessed with CT peritoneography has not been reported. Radiologic response to IP chemotherapy was reported in a series of 11 ovarian carcinoma patients stratified into three categories by distribution of intraperitoneal Tc-99m and was suggestive of better response in patients with free-flowing infusate than in those with loculation, but overall survival was not reported.^21^ However, this study relied on the subjective assessment of scans and division into three arbitrarily defined categories.

Malignant peritoneal mesothelioma (MPM) is diagnosed in approximately 250 patients in the United States per year and is generally linked to asbestos or, less commonly, radiation exposure.^22^,^23^ Until very late in its natural history, the disease generally spreads superficially over the peritoneal surface, rarely metastasizing outside the abdomen.^24^ Mesothelioma can be classified as epithelioid, sarcomatoid, or biphasic based on histological appearance. Epithelioid subtype, female sex, and younger age have been associated with better outcomes in numerous treatment and epidemiologic contexts.^25^–^28^

We have reported previously on the treatment of MPM with a combination of surgical debulking with intraoperative heated intraperitoneal chemotherapy and catheter-administered outpatient IP chemotherapy, including cisplatin dosed by body surface area (BSA) at 100 mg/m^2^.^28^,^29^ Initial debulking surgery before IP chemotherapy was performed with a goal of removing all tumor nodules greater than 0.5 cm in depth or plaques greater than 0.5 cm in diameter, because residual disease greater than 0.5 cm has been associated with adverse outcomes in peritoneal carcinomatosis, in general, and MPM, in particular.^26^,^27^,^30^ As a standard assessment of catheter function and infusate distribution, many of these patients underwent CT peritoneography.^31^ The purpose of the current study was to determine whether objective, quantitative parameters determined from CT peritoneograms were associated with overall survival and/or complications as manifested by posttreatment glomerular filtration rate (GFR) in patients treated with cisplatin-based IP chemotherapy.

## Patients and Methods

### Patients

Our institutional review board (IRB)-approved protocol for the treatment of MPM has been previously reported.^28^,^29^ Retrospective chart review identified 38 patients who underwent CT peritoneography while receiving IP chemotherapy between February 2000 and August 2011. Baseline characteristics of the 38 patients are reported in Table 1.

**Table 1:** Baseline characteristics

Characteristic	Patients (*N* = 38)
Female sex	19 [50 %]
Age (years), median [range]	61 [21–83]
Body surface area (m^2^), mean [SD]	1.92 [0.25]
Histologic subtype	
Epithelioid	34 [89 %]
Biphasic	4 [11 %]
Residual disease >0.5 cm	10 [26 %]

*SD* standard deviation

To examine the relationship between BSA and systemic cisplatin levels, on an IRB-approved protocol, blood was collected during seven hyperthermic intraoperative intraperitoneal chemotherapy (HIPEC) cases.

### Imaging and Computer-Aided Volume and Surface Area Quantification

After injection of between 100 and 500 cc of diluted iohexol contrast into IP catheters with patients in supine or semi-Fowler position, patients underwent standard abdominopelvic CT scans. Smaller volumes of contrast were used in patients who experienced pain or pressure with injection. CT scans were performed with patients in the supine position. Contrast-filled compartments are identifiable based on higher density than surrounding structures on CT images. An in-house segmentation algorithm was developed and applied to assist in calculating volumes and surface areas of the compartments in this work.

We manually selected a region-of-interest (ROI) enclosing all contrast-filled compartments on a single image. Localization followed by segmentation of each of the compartments inside the ROI was then performed automatically by the developed algorithm. Once the segmentation was completed on an image, the result was propagated to neighboring images, with automatic segmentation of the contrast-filled compartments. This process continued iteratively until all compartments were segmented. To ensure correct results, computer-generated compartment contours were superimposed on the original images for inspection and modification as needed by a radiologist.

Once the segmentation was finalized, volumes and surface areas of the compartments were automatically calculated. The compartment volume was calculated by multiplying the total number of all compartments’ voxels and the image resolutions along x—(in-plane), y—(in-plane), and z—(axial) directions. The compartment surface area was defined as the sum of the interface areas of all compartment voxel sides facing noncompartment voxels, where the area of a voxel side is calculated by multiplying the image resolutions along the two directions spanning the plane at which the voxel side resides.

The computer algorithms and a number of manual interaction functions, such as selection of ROI and modification of suboptimal computer results, were integrated into a user-friendly image-viewing system developed with the Matlab computer language by the research group.

### Cisplatin Pharmacokinetics

Blood plasma samples were diluted 1:100 in 2 % HNO_3_, 1 % methanol, 0.2 % Triton 100-X solution and analyzed for platinum concentrations using a Perkin-Elmer Elan DRC II (Perkin Elmer, Shelton, CT) Inductively Coupled Plasma Spectrophotometer (ICP-MS) equipped with an AS 93+ autosampler. The platinum concentration of calibration standards was chosen to cover the expected range of platinum concentrations in the diluted plasma samples: 1, 5, and 10 μg/L. Matrix-induced interferences were corrected using an iridium internal standard to match the mass and ionization properties of the platinum. Stock internal standard spiking solution was prepared and added to all calibrators and samples in the same concentration: 50 ng iridium per tube. After the initial instrument calibration, quality control samples (QC-plasma spiked in our laboratory and serum samples of known platinum concentration provided by Institut de Sante Publique du Quebec) were run. To control instrument drift over the period of running hours, we ran QC samples every 10–15 samples, and recalibrated if QCs did not meet quality control criteria (±10 % of target values). For the 60-min duration of HIPEC (samples at 10, 30, and 60 min), cisplatin plasma AUC was calculated by Trapezoidal Rule.

### Statistical Analysis

All statistical analysis was performed using SAS Version 9.2. The LIFETEST procedure was used to produce the Kaplan–Meier survival estimates for all patients and to compare survival by volume of residual disease (>0.5 vs. <0.5 cm). To determine whether the presence of bulky disease was independent of peritoneogram parameters and GFR, the TTEST procedure was used to test for differences in mean surface area and volume of the contrast-filled compartment as well as pre- and posttreatment GFR between patients with residual disease >0.5 cm versus those with residual disease <0.5 cm after initial tumor debulking surgery. Univariate Cox models were conducted using the PHREG procedure for survival. In addition to the surface area and volume of the contrast-filled compartment, any covariate with a *p* value < 0.1 in the univariate analysis was selected for multivariate analysis. Overall survival was measured from IP catheter placement.

Linear regression analyses with posttreatment GFR as the outcome measure were conducted using the REG procedure. The regression models included pretreatment GFR, BSA (since cisplatin is dosed based on BSA), the surface area and volume of the contrast-filled compartment, and two-way interactions between BSA, surface area or volume, with only statistically significant (*p* < 0.05) two-way interaction terms retained in the final model. Residual disease status was not included, as it was not statistically significant in univariate or multivariate models. Pre- and posttreatment GFR were calculated from, respectively, the last serum creatinine measured before IP catheter placement and the first serum creatinine measured after IP catheter removal, by Cockgroft-Gault formula.^32^ BSA was calculated from the height and weight at the time of IP catheter placement by Mosteller formula.^33^

Three patients underwent CT peritoneography twice. For these patients, we used the mean surface area and volume of the contrast-filled compartment from the two CT peritoneograms.

For HIPEC pharmacokinetic data, linear regression with cisplatin plasma AUC as the outcome measure and BSA as the independent variable was conducted using the REG procedure.

## Results

**Fig. 1:**
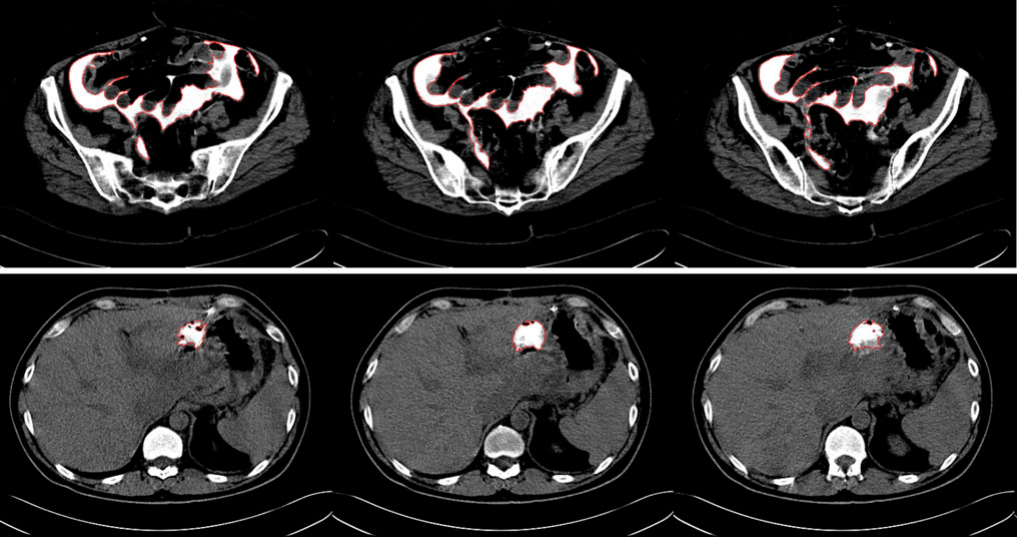
Computer**-**aided quantitative peritoneogram images. The contrast-filled compartments are outlined in *red* for **a** well-distributed and **b** loculated intraperitoneal contrast

Examples of computer-aided peritoneogram analysis images are presented in Fig. 1. Median overall survival by Kaplan–Meier analysis, pretreatment and posttreatment GFR and computer-aided peritoneogram volume and surface area data are presented in Table 2. There were no statistically significant differences in volume or surface area parameters between patients with residual disease >0.5 cm versus those with residual disease <0.5 cm after initial debulking. We therefore considered the peritoneogram parameters independent of the volume of residual disease.

**Table 2:** Overall survival, GFR, and CT peritoneography parameters

Parameter	Overall	Residual disease < 0.5 cm	Residual disease > 0.5 cm	*p* value
Overall survival (mo), median [95 % CI]	48 [11–76]	62 [47–94]	5 [1–22]	<0.0001
Pretreatment GFR (cc/min), mean [SD]	96 [35.5]	101.9 [38.5]	79.5 [17.9]	0.0872
Posttreatment GFR (cc/min), mean [SD]	90.1 [42.6]	96.2 [46.4]	73.1 [24.1]	0.1444
Contrast-filled compartment volume (cm^3^), mean [SD]	558.4 [532]	582.5 [458.4]	491.0 [725.6]	0.6468
Contrast-filled compartment surface area (cm^2^), mean [SD]	1261.7 [1158.5]	1405 [1216.8]	860.4 [912.7]	0.2062

Patient outcomes following intraperitoneal chemotherapy and algorithm-derived peritoneogram values

*GFR* glomerular filtration rate, *CT* computed tomography, *CI* confidence interval, *SD* standard deviation

We used univariate Cox models to determine which covariates to include with volume and surface area in the multivariate Cox model of overall survival. Four variables (age, sex, histologic subtype, and residual disease >0.5 cm) had *p* < 0.1 and were included in the multivariate model. We found that, controlling for age, sex, histologic subtype, and residual disease, the surface area of the contrast-filled compartment had a positive relationship with overall survival (*p* = 0.0201) and the volume of the contrast-filled compartment had a negative relationship with overall survival (*p* = 0.0341; Table 3). In terms of proportional hazards, controlling for the above covariates, a 1 standard deviation increase in surface area is predicted to result in a hazard ratio of 0.222 (95 % confidence interval, 0.063–0.79) and a 1 standard deviation increase in volume is predicted to result in a hazard ratio of 3.165 (95 % confidence interval, 1.09–9.193).

**Table 3:** Cox models using overall survival as outcome

Covariate (univariate model)	Hazard ratio	95 % CI	*p* value
Age (years)	1.038	0.998–1.079	0.0628
Sex (female vs. male)	0.319	0.119–0.858	0.0235
Body surface area (m^2^)	1.160	0.179–7.539	0.8764
Histologic subtype (biphasic vs. epithelioid)	20.798	4.419–97.89	0.0001
Residual disease (>0.5 vs. <0.5 cm)	11.685	3.785–36.074	<0.0001
Contrast-filled compartment volume (cm^3^)	1.000	0.999–1.001	0.3551
Contrast-filled compartment surface area (cm^2^)	1.000	0.999–1.000	0.0907

Covariate (multivariate model)	Hazard ratio	95 % CI	*p* value
Age (years)	1.06	1.002–1.12	0.0424
Sex (female vs. male)	1.188	0.347–4.066	0.7835
Histologic subtype (biphasic vs. epithelioid)	4.502	0.81–25.026	0.0856
Residual disease (>0.5 vs. <0.5 cm)	7.657	1.991–29.456	0.0031
Contrast-filled compartment volume (cm^3^)	1.002	1.000–1.004	0.0341
Contrast-filled compartment surface area (cm^2^)	0.999	0.998–1.000	0.0201
Overall model	–	–	<0.0001

All variables with *p* < 0.1 in the univariate analysis were included in the multivariate model

Overall survival was measured from the time of intraperitoneal catheter placement

*CI* confidence interval

**Fig. 2:**
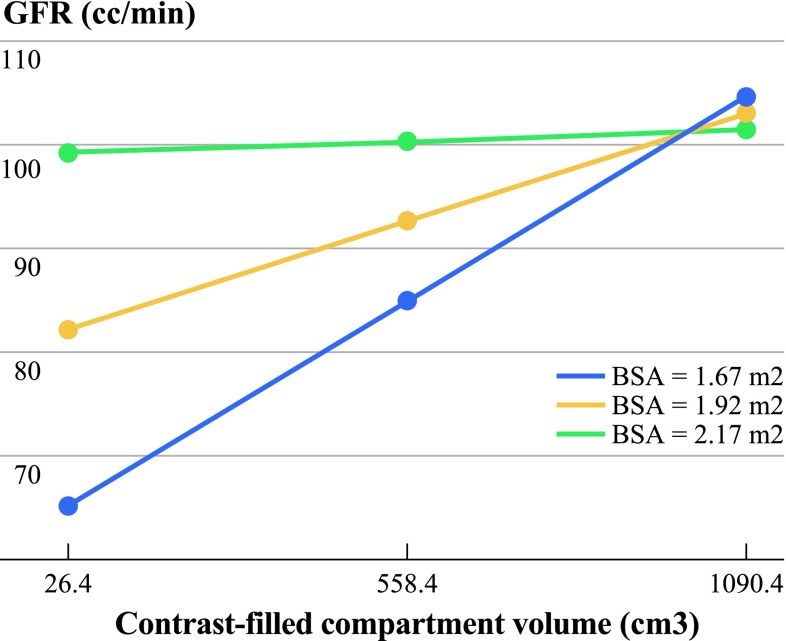
Predicted posttreatment glomerular filtration rate (GFR). For a patient with a pretreatment GFR of 100 cc/min and a contrast-filled compartment surface area set at the sample mean (1,262 cm^2^), comparing body surface area set at the sample mean ± one standard deviation (low, average, and high BSA) and contrast-filled compartment volume set at the sample mean ± one standard deviation (low, average, and high volume)

We used linear regression with posttreatment GFR as the outcome and included pretreatment GFR and BSA, along with volume and surface area as covariates, as well as the two-way interaction between volume and BSA (the only two-way interaction to reach statistical significance). We found that, controlling for pretreatment GFR, BSA, surface area, and the interaction between volume and BSA, the volume of the contrast-filled compartment had a statistically significant positive relationship with posttreatment GFR (*p* = 0.0167; Table 4). The interaction between volume and BSA is illustrated in Fig. 2.

**Table 4:** Final linear regression model using posttreatment GFR (cc/min) as outcome

Covariate (linear regression model)	Estimated regression coefficient	*p* value
Pretreatment GFR (cc/min)	0.802	<0.0001
Body surface area (m^2^)	69.969	0.0182
Contrast-filled compartment volume (cm^3^)	0.154	0.0167
Contrast-filled compartment surface area (cm^2^)	−0.003	0.5893
Interaction between volume and body surface area	−0.07	0.026
Overall model	–	<0.0001

Multivariate linear regression model, including only those two-way interactions with *p* < 0.05

*GFR* glomerular filtration rate

To validate whether the effect of BSA on posttreatment GFR was related to systemic cisplatin levels, we analyzed the relationship between BSA and cisplatin plasma AUC in seven patients undergoing HIPEC using linear regression. We found that higher BSA was associated with lower plasma AUC during HIPEC (estimated regression coefficient = −89.7 mg/min/L/m^2^, *p* = 0.0381).

## Discussion

Our data suggest that larger surface areas of the compartment available to chemotherapy administered by IP catheters are associated with improved overall survival in MPM patients. This is consistent with the rationale for IP treatment of peritoneal surface-spreading malignancies: direct drug contact with a larger peritoneal surface area means that more drug is directly delivered to potential areas of tumor spread.^2^ Controlling for surface area, larger volumes were associated with decreased survival, suggesting that a high surface area-to-volume ratio of the contrast-filled compartment is optimal. This is consistent with the observation that loculated intraperitoneal compartments are more spherical, whereas free-flowing intraperitoneal compartments have irregular edges corresponding to the peritoneal organs, notably the small bowel. In addition, a higher surface area-to-volume ratio ensures that a larger proportion of the infused chemotherapy is in close proximity to peritoneal surfaces.

In the final multivariate Cox model, in addition to larger surface area and smaller volume, younger age and residual disease <0.5 cm were associated with improved overall survival. Indeed, these factors, as well as histology and sex, have been identified as prognostic factors in previous studies.^25^–^28^ Peritoneography does not outweigh these factors but is valuable in predicting response of patients to intraperitoneal therapy, given the other prognostic information associated with each of these factors. In this analysis, histology and sex were not statistically significant predictors of overall survival, which may be attributable to the fact that in our cohort all of the females had epithelioid disease and 18 of 19 females had no residual disease >0.5 cm, whereas all 4 patients with biphasic disease also had residual disease >0.5 cm.

Statistical analysis showed no differences in measured peritoneogram volume or surface area between patients with residual disease >0.5 vs. <0.5 cm, making it likely that the peritoneogram parameters were independent of observed tumor volume. We therefore included both groups of patients in the survival analysis. Indeed, the final multivariate Cox model showed that the volume and surface area of the contrast-filled compartments, and the presence of residual disease >0.5 cm were all statistically significant independent predictors of overall survival. However, the relatively small number of patients with residual disease >0.5 cm limits our ability to draw conclusions about this subgroup.

Our data suggest that larger volumes of the compartment available to IP catheter-administered chemotherapy are associated with higher posttreatment GFR in MPM patients, which is consistent with the physiology of the peritoneal diffusion barrier. Elevated intra-abdominal pressure is associated with increased fluid transfer from the peritoneal space; the major diffusion barrier is the blood vessel wall and surrounding interstitium, rather than the anatomic peritoneum.^34^ Although we have not directly measured intra-abdominal pressures, it is possible that increased compartment volume are associated with lower compartmental pressures, resulting in lower intravascular drug levels and less cisplatin nephrotoxicity.

In the final multiple linear regression model, larger BSA was associated with higher posttreatment GFR, possibly because of lower systemic drug exposure. This is consistent with our HIPEC pharmacokinetic data (in which free flow is assured, as chemoperfusion occurs during surgery, before adhesions can form), which showed that higher BSA was associated with lower cisplatin plasma AUC.

The strongest clinical evidence for improved survival with catheter-administered IP chemotherapy is in advanced ovarian carcinoma, including a large meta-analysis suggesting improved overall and disease-free survival for patients who receive IP chemotherapy.^35^ The landmark GOG-172 trial for ovarian carcinoma reported a significant difference in overall survival for patients receiving intraperitoneal chemotherapy versus intravenous chemotherapy (median overall survival 65.6 vs. 49.7 months, *p* = 0.03 by intention to treat analysis); however, only 42 % of those assigned to intraperitoneal chemotherapy completed all six cycles, due chiefly to catheter-related complications, as well as renal/metabolic toxicities, neuropathy, and nausea/vomiting/dehydration.^36^,^37^ Prognostic factors, not only of overall survival but of potential chemotherapy-related toxicities, are needed to optimally plan IP chemotherapy, given the high rate of discontinuation due to adverse events.

Our retrospective data suggest that quantitative CT peritoneography provides parameters associated with overall survival (compartment surface area and volume) and posttreatment GFR (compartment volume) in MPM patients undergoing IP chemotherapy. In clinical practice, we therefore would recommend routine CT peritoneography on all patients undergoing IP chemotherapy to confirm catheter function and to assess drug distribution subjectively. This will provide valuable prognostic information and may affect management, because patients with poor distribution may be best served by systemic chemotherapy in addition to or instead of IP chemotherapy to improve their overall survival.

It is possible that our results reflect a selection bias in which patients might have been chosen to undergo CT peritoneography because of clinical suspicion of catheter-related complications. In addition, patients who experienced pain or pressure with injection received lower volumes of contrast. It is likely that these symptoms indicated that the volume available to intraperitoneal contrast was filled, but use of a standardized volume for all patients would provide added validity. Finally, standard prone-position CT scans were used; however, they may not have reflected the physiologic distribution of intraperitoneal chemotherapy for different body positions. Prospective studies should be undertaken, using a standardized contrast volume with patients in multiple positions, to confirm the prognostic value of CT peritoneography and to extend our findings to other diseases including advanced ovarian carcinoma.
